# Innovative Applications of *Tenebrio molitor* Larvae in the Production of Sustainable Meat Sausages: Quality and Safety Aspects

**DOI:** 10.3390/foods13101451

**Published:** 2024-05-08

**Authors:** Agnė Jankauskienė, Sandra Kiseliovienė, Dominykas Aleknavičius, Ieva Miliūnaitė, Sigita Kerzienė, Žydrūnė Gaižauskaitė, Ignė Juknienė, Paulina Zaviztanavičiūtė, Aistė Kabašinskienė

**Affiliations:** 1 Department of Food Safety and Quality, Veterinary Academy, Lithuanian University of Health Sciences, Tilzes St. 18, LT-47181 Kaunas, Lithuania; agne.jankauskiene@lsmu.lt (A.J.); ieva.miliunaite@stud.lsmu.lt (I.M.); igne.jukniene@lsmu.lt (I.J.); paulina.zavistanaviciute@lsmu.lt (P.Z.); 2 Food Institute, Kaunas University of Technology, Radvilenu pl. 19, LT-50254 Kaunas, Lithuania; sandra.kiselioviene@ktu.lt (S.K.); zydrune.gaizauskaite@ktu.lt (Ž.G.); 3 Divaks, UAB, Vinco Kudirkos g. 22-12, LT-01113 Vilnius, Lithuania; dominykas@divaks.com; 4 Department of Physics, Mathematics and Biophysics, Veterinary Academy, Lithuanian University of Health Sciences, Tilzes St. 18, LT-47181 Kaunas, Lithuania; sigita.kerziene@lsmu.lt

**Keywords:** biogenic amines, nitrate, nitrite, amino acids, fatty acids, by-products, volatile fatty acids, peroxides

## Abstract

With the world’s population continuing to grow, ensuring sustainable protein sources for everyone is becoming increasingly challenging. Despite meat being considered unsustainable, people find it challenging to abstain from consuming it. However, one solution to this dilemma could be the incorporation of mealworms into conventional meat products, i.e., sausages. The incorporation of mealworms into sausage formulations appears to shift the fatty acid profile towards higher levels of monounsaturated fats and polyunsaturated fatty acids (PUFAs), particularly omega-3s, potentially enhancing the nutritional value and offering health benefits. Therefore, our study aimed to improve the nutritional value and safety parameters of traditional sausages by enriching them with the flour of mealworm larvae. For this purpose, the larvae were reared on a sustainable substrate with brewery by-products, brewer’s yeast, and carrots. They were used frozen and freeze-dried in sausage recipes, replacing pork in different proportions. The analysis of the product’s chemical safety parameters (biogenic amines, nitrates and nitrites, volatile fatty acids (FA), and peroxide) and nutritional value (including collagen, cholesterol, amino acids, FA, and hydroxyproline) was carried out in an accredited laboratory. The results of our study have demonstrated that the incorporation of mealworms into sausages, particularly through freeze-drying, increased fat content and enhanced the profile of FA, including omega-3s while reducing protein and cholesterol levels, and altering collagen content, suggesting improved nutritional value and potential health benefits without compromising the safety of the product. Therefore, we are highlighting that the addition of mealworms influences the quality of amino acids positively and maintains biogenic amine levels within safe limits, alongside a negligible impact on nitrates and nitrites and a reduction in peroxide values. These findings indicate an overall improvement in sausage quality and safety without compromising safety.

## 1. Introduction

The world’s population continues to grow and despite recent slower growth of the population in developed countries [1], the general trend is predicted that by 2030, the world’s population will reach 8.5 billion, by 2050—9.7 billion, and by 2100—10.4 billion [2]. Approximately one billion people consume insufficient amounts of protein [3,4]. Moreover, it is important to note that not all proteins contain all essential amino acids [4]. The challenge of preventing hunger and malnutrition will increase further. As the population expands, so does the number of individuals lacking access to high-quality proteins. However, the escalating demand for beef, dairy products, and pork as primary protein sources will significantly exacerbate the negative impacts of climate change. Anthropogenic climate change primarily results from the release of greenhouse gases, including carbon dioxide, methane, and nitrous oxide, with agriculture playing a significant role in the emissions of the latter two gases [5]. The environmental impact varies greatly across different food items of different origins when considering greenhouse gas emissions from production to consumption. Protein-rich foods, such as legumes, meat, fish, cheese, and eggs, exhibit substantial differences, with emissions per kilogram ranging by a factor of 30. Legumes, poultry, and eggs have the lowest emissions, while beef, cheese, and pork have the highest [6]. Conventional livestock farming today causes significant ecological damage, such as greenhouse gas emissions, land acidification, soil nitrification and erosion, eutrophication, biodiversity loss, and global freshwater stress [7]. However, reduction in climate change can be achieved not only through the promotion of plant-based food production but more sustainable sources of animal protein, such as the cultivation of the larvae, *Tenebrio molitor* (Linnaeus, 1758), which exhibit particularly high efficiency [8]. In the future, more food will have to be produced on less land, just as greenhouse gas emissions must be reduced. In this context, mealworms represent a sustainable and resource-efficient option for protein production in circular food systems [9]. They are an efficient biomass converter for low-quality by-products such as spent grains from breweries. The valorization of agricultural and industrial by-products with insects is an increasingly researched strategy. Studies show that it is more efficient to obtain protein from mealworms than from other traditional farm animals in terms of freshwater resources [10,11]. Mealworm farming is cost-effective, energy-efficient, has a low ecological footprint, and one of the most important species for converting plant biomass into high-quality proteins [12,13]. Therefore, mealworms can be perfectly adapted to the principles of the circular economy. Our previous studies have shown that the nutritional value of mealworms is not inferior to other protein sources such as soy, fish, and meat [14]. Mealworms are rich not only in high protein content but also in essential amino acids, as well as having a favorable FA profile, especially in terms of PUFAs, and high fiber content [14,15]. 

Although entomophagy (insect consumption for food) is slowly gaining more visibility in the Western world, it often elicits consumer acceptance only on a trial basis, in part due to the desire for adventure, environmental protection, health benefits, or a combination thereof, but does not become part of the diet [16]. According to the researchers, the Western public’s opinion about edible insects would change if not only a lot of safety research were conducted, but also the image of the food would be in an acceptable form, e.g., embedded in already accepted products such as sausages, bread, cocktails, and the like [16,17,18]. 

Attempts to integrate mealworms into meat products have already been made; however, most researchers were focusing on technological aspects, and we did not find a study that also examined chemical safety. Previous studies, such as that of Hyun-Wook et al., showed that the addition of mealworms to sausages increased the output and firmness of emulsion sausages [19]. According to Vlahova-Vangelova et al., the suitable pH of edible insect meal flour, the stable emulsions formed, and the good water-binding capacity both before and after cooking confirmed the potential of a suitable meat substitute in the meat industry [20]. Talens et al. also see a successful way to add up to 50 percent of edible insect flour to sausages, which almost does not change the taste characteristics [21]. 

However, it should be noted that, like all ingredients in food, it may not be beneficial for everyone, perhaps even harmful, and have certain limitations. Various drying methods, including rack oven drying, vacuum drying, and freeze-drying, have the potential to alter the color of larvae and the profiles of volatile compounds associated with Maillard reactions [22]. Moreover, the industrial procedures employed in producing mealworm powders may impact several aspects of the final product, such as its physical and physicochemical properties, color, and morphological characteristics. These alterations can result in differing perceptions regarding the product’s appearance, flavor, texture, and overall acceptance [23]. Furthermore, further investigation is needed regarding the allergenicity of this species, as mealworms, like other insects, have the potential to trigger allergies in individuals who are already allergic to crustaceans and dust mites [24,25,26]. 

Overall, this study aimed to investigate the feasibility of incorporating mealworms into sausage formulations as a sustainable protein source rich in trace elements and essential amino acids with a perfect ratio of omega 3 and 6, while ensuring sausage quality and safety. The findings contribute to the growing area of research on insect-based foods and their potential role in addressing global food security and sustainability challenges. Therefore, the tasks of our research were to grow mealworms using grain, brewer’s yeast used by breweries, and carrots as sustainable substrates, to use the flour of mealworm larvae to partially replace pork in sausages, and to examine the chemical safety of the sausages. We propose that substituting pork with mealworms in sausages may alter their technological properties, yet the safety parameters should remain unchanged. 

## 2. Materials and Methods

### 2.1. Insect Rearing and Samples Preparation

The cultivation conditions and substrates for mealworms were selected based on previous research, prioritizing larvae grown with dehydrated brewers’ spent grain due to their highest amount of trace elements, the most protein, the best sensory evaluation, excluding the control, the highest fiber content, and the best FA and amino acid composition [14]. 

The yellow mealworm larvae were raised under controlled conditions at the Divaks company’s insect research and development facility in Vilnius, Lithuania [27], with a temperature of 27 ± 2 °C, humidity of 60 ± 5%, and lighting provided for at least 1 h per day. The eggs were obtained from adult beetles of various age groups, using wheat bran from Fasma, Lithuania [28], as the egg-laying substrate, and carrots for moisture. Approximately 30,000 individuals, equivalent to 17 g of eggs, were placed in containers with 1.5 kg of dry feed, consisting of dehydrated brewers’ spent grain from Eurokorma, Lithuania [29] and brewer’s yeast from Ekoproduktas, Lithuania [30]. Wet feed, including approximately 3.45 kg of carrots from Sanitex, Lithuania [31], was provided three times a week, with a dry feed to brewer’s yeast ratio of 9:1. The larvae were considered fully grown upon the appearance of the first pupae after 56 days of growth. After sifting to remove waste and remnants, the larvae underwent a 24 h fasting period in a climate chamber before processing. They were then frozen at −18 °C and subjected to subsequent analysis.

Some mealworms were dried in a thermostat at 103 °C until reaching a constant mass, while another portion underwent rapid freezing at −35 °C for 8 h using a Liebherr fast freezer. Freeze-drying is performed in a lyophilized atmosphere until reaching 80 °C under a pressure of 73 (Pa), lasting a total of 72 h. The lyophilized and dried larvae were then milled using a laboratory-scale mill at 6000 rpm.

### 2.2. Sausage Preparation, Processing and Coding

Lean pork and back fat were selected from Cesta, Lithuania [32] and delivered to the laboratory within 72 h after slaughtering in plastic containers at a temperature of 4 °C (Table 1). Pepper, salt, and ice were purchased from Sanitex, Lithuania [31]. Natural pork intestines purchased fresh from Cesta, Lithuania, were utilized for sausage casing. 

All components were thoroughly mixed and transferred into metal forms. Subsequently, the mixture underwent heating in an oven at 110–120 °C until reaching a core temperature of 75 °C, a process monitored by inserting a digital thermometer, which typically took 50 to 65 min. The sausages were produced in three independent repeats, with the same batch of insect larvae used in each repeat.

### 2.3. Methods of Determining Physicochemical Parameters

#### 2.3.1. Determination of Acidity (pH)

The pH levels of the samples were assessed following the standard procedure outlined in EN ISO 2917:2002 [33]. A pH meter (Inolab 3, Hanna Instruments, Milano, Italy) was utilized for the evaluation. Before analysis, the pH meter underwent calibration at two reference points, pH 4.01 and 7.00, employing standard buffers (Sigma Aldrich, Saint Louis, MO, USA). The pH electrode was immersed in a mixture of sausages and water (1:1) for the experimental preparation of samples, comprising both larvae and substrate.

#### 2.3.2. Cooking Loss

Cooking loss was calculated analogously to Scholliers et al. in the study conducted [34]. Cooking losses were determined immediately before baking and after heat treatment after the samples had cooled to a temperature of 4 degrees. 

#### 2.3.3. Texture

Before texture analysis, the sausages were sliced to a thickness of 6 mm. The hardness was determined by applying a maximum compression force using the Stevens-LFRA Texture Analyzer (Voland Corp., New York, NY, USA), with a 10-mm-diameter plunger and a compression rate of 2 mm/s at 60% compression.

#### 2.3.4. Method for Determining Moisture Content

The moisture content was assessed using the reference method 1442:2023, outlined for the determination of moisture content in meat and meat products [35]. The procedure involved a series of heating, cooling, and weighing cycles, repeated iteratively until the discrepancy between the results of two successive weightings following 1 h of heating did not exceed 0.1% of the sample mass.

### 2.4. Nutritional Value

The tests of nutritional value and safety parameters were carried out in an accredited laboratory: Chemical Science Laboratory, Food Institute, Kaunas University of Technology, Lithuania [36]. 

#### 2.4.1. Determination of Fat Content

The determination of fat content followed the standard ISO 1443:2000 “Meat and meat products—Determination of total fat content” [37]. 

#### 2.4.2. Determination of Salt Content

To determine the salt content, 3 g of finely ground sausages were weighed and placed into a 200 mL beaker, followed by the addition of 100 mL of distilled water. The mixture was thoroughly stirred with a glass rod equipped with a rubber tip for 10 min to ensure the dissolution of salt and prevent larger larvae or substrate particles from remaining. Subsequently, the mixture was allowed to settle for 5 min. A 15 mL portion of the settled liquid was withdrawn using a pipette and titrated with 0.01 N silver nitrate solution, employing potassium chromate solution as an indicator. The percentage of table sodium chloride in the product under investigation was determined using the formula:

x = 0.0029 × v × 100 × 100/b × g(1)

where v represents the volume of 0.05 N silver nitrate solution used for titration in mL, g denotes the amount of ground larvae or substrates taken for the study in grams, and 0.05 N signifies the titer of the silver nitrate solution.

#### 2.4.3. Determination of the Total Ash Content in Sausages

Ash content was determined by dry ashing in a furnace oven at 550 °C for 5 h. The findings were documented with a precision of 0.01%. Method repeatability was ensured; therefore it must not surpass the calculated value of r. The r value was determined by the formula:

r = 0.0990% + 0.00933 w(2)

where w—an average of two results, expressed as a percentage.

#### 2.4.4. Collagen and Hydroxyproline Content in Sausages

The amounts of collagen and hydroxyproline in experimental sausages were determined according to the standard: LST ISO 3496:2001, meat and meat products determination of hydroxyproline content [38]. 

#### 2.4.5. Cholesterol Determination Method

The cholesterol content of the samples was evaluated by the high-performance liquid chromatography method (HPLC system Shimadzu Corp., Kyoto, Japan, with UV/VIS detector SPD-20A). Reversed-phase column YMC-Pack ODS-A (YMC Co., Ltd., Kyoto, Japan), 150 × 4.0 mm, I.D, 12 nm, s—5 µm, was used for analysis. Working conditions: mobile phase flow rate—1.2 mL/min; injection volume 20 µL; column temperature 30 °C; detector measurement wavelength—205 nm; elution—isocratic; the mobile phase was a mixture of acetonitrile and methanol (70:30 V/V). Preparation of a test sample solution. Approximately 0.25 g of the test sample was placed in 5 mL of 2% KOH in ethanol and heated in a water bath at a temperature of 50 °C. After heating for 2 h, the saponified mixture was cooled in a stream of running water to 20 °C and 5 mL of distilled water was added. Cholesterol was extracted by shaking vigorously, adding 10 mL of hexane twice. The hexane fraction was collected by evaporating it with nitrogen until it reached 3 mL. The residue was dissolved in 3 mL of a mixture of acetonitrile: methanol (70:30, V/V). The mixture was filtered through a membrane filter with a pore size of 0.45 µm and analyzed. 

The cholesterol content of food products was determined according to the following formula:

(3) $$amount\;of\;analite\;\left(mg/100g\right)=\frac{M_{ch}\cdot S_{ch}\cdot V_{1}\cdot V_{4}\cdot m_{1}}{S_{st}\cdot V_{3}\cdot V_{2}\cdot m_{2}}$$

where *M_ch_*—mass of cholesterol standard, in injection volume, mg; *S_ch_*—cholesterol peak area in the sample; *V*_1_—volume of hexane fraction taken for evaporation, µL; *V*_2_—amount of hexane required for extraction, mL; *m*_1_—mass to which cholesterol content is converted, g (here 100 g); *S_st_*—peak area of cholesterol standard; *V*_3_—the volume of the analyzed sample (here 20 µL); *V*_4_—the volume of the mobile phase in which the residue after evaporation with nitrogen is dissolved, *m*_1_; *m*_2_—mass of the sample taken for analysis, g.

#### 2.4.6. Method for Determination of Amino Acids

Amino acid analysis was conducted following the guidelines outlined in commission regulation (EC) No. 152/2009 of 27 January 2009, which specifies the methods for sampling and analysis for the official control of feed [39]. The hydrolysis of the samples adhered to the procedures delineated in Commission Regulation No. 152/2009. In summary, approximately 100 mg of the sample underwent hydrolysis with a 6 M HCl solution containing 0.1% *w/v* phenol in a laboratory oven at 110 ℃ for 23 h. Following hydrolysis, the resulting mixture was cooled, pH adjusted to 2.2, and diluted to 250 mL with citrate buffer (containing 0.1% *w/v* phenol and 5% *v/v* thiodiglycol). The resulting sample solution was utilized for derivatization. Concentrations of amino acids were determined using a GCMS-QP2010 (Shimadzu, Japan) gas chromatograph coupled with a mass spectrometer. Individual analyte concentrations were determined using a calibration curve. To 50 µL of the sample solution, 50 µL of internal standard (~500 µM of norleucine), 120 µL of 0.1 M HCl, 40 µL of 2 M NaOH, 200 µL of a methanol-pyridine mixture (MeOH: Pyridine—4:1), and 500 µL of chloroform were added for derivatization. Derivatization was initiated with 50 µL of isobutyl chloroformate. Subsequently, 40 µL of 12.5 M NaOH was added, and the mixture was rederivatized with 50 µL of iso-butyl chloroformate. The resultant mixture was centrifuged at 13.2 krpm, and the organic layer was dried with anhydrous sodium sulfate before analysis. For analysis, a Capillary Rxi^®^-5MS column (Restek, Bellefonte, PA, USA) (30 m in length, coating thickness of 0.25 µm, inner diameter of 0.25 mm) was employed. The mass spectrometer operated in single ion monitoring mode, with analyte injection in splitless mode. Operating parameters were as follows: MS ion source temperature: 220 °C, MS interface temperature: 300 °C, helium (carrier gas) flow: 0.99 mL/min, injector temperature: 250 °C, oven temperature program: 100 °C (held for 0.5 min), ramped at 10 °C/min to 310 °C (held for 4 min).

#### 2.4.7. Method for Determination of FA

The analysis of FA composition was conducted according to established methodologies. Sample preparation adhered to the protocols outlined in the standard LST EN ISO 12966-2:2017 Part 2 [40], which delineates the procedures for methyl ester preparation of FAs. FA methyl esters were analyzed using a gas chromatograph GC-MS (PerkinElmer Clarus 680) coupled with a mass spectrometer PerkinElmer Clarus SQ8T. The chromatographic column temperature was initially set at 60 °C for 1 min, followed by a linear increase of 12 °C per minute until reaching 250 °C, where it was held for 10 min. The spectrometer temperature ramped up at a rate of 5 °C per minute to 300 °C, remaining constant for 20 min. The evaporator temperature was maintained at 250 °C. Calibration curves for this analysis were established using the standard Supelco 37 Component FAME Mix provided by Merck & Co., Inc. (Rahway, NJ, USA).

### 2.5. Safety Parameters

#### 2.5.1. Biogenic Amines

Biogenic amines were determined analogously to Jankauskienė et al.’s previously published article [41]. 

#### 2.5.2. Determination of Nitrite and Nitrate Content in Mealworms and Sausages

The investigation of nitrite content was conducted following the method delineated in ISO 2918:1975, titled “Meat and meat products—Determination of nitrite content‘’ [42]. Meanwhile, the examination of nitrate was carried out following the methodology specified in ISO 3091:1975, titled “Meat and meat products—Determination of nitrate content’’ [43].

#### 2.5.3. Method for Determination of Peroxide Content

The peroxide value was assessed according to the ISO 27107:2010 standard, which pertains to the determination of peroxide value in animal and vegetable fats and oils through potentiometric endpoint determination [44]. 

#### 2.5.4. Determination of Volatile FA Content

The determination of volatile FA content followed the guidelines set forth by the minister of agriculture of the Republic of Lithuania, as outlined in the technical regulations for the assessment of meat and poultry freshness (FMAP) [44]. These regulations were developed in compliance with the European Parliament and Council Regulation (EC) No. 853/2004, dated April 29, 2004, which establishes specific hygiene requirements for foodstuffs of animal origin (OJ 2004 special edition, chapter 3, volume 45, p. 14) [45], as last amended in 2012 by Commission Regulation (EU) No. 16/2012, dated 11 January 2012 (OJ 2012 L 8, p. 29) [46]. For the analysis, 25 g of milled sausages were placed into a 0.75–1.0-L capacity round-bottomed flask, to which 150 milliliters of a 2% sulfuric acid solution were added. The flask contents were stirred, tightly sealed, and subjected to steam distillation until 200 milliliters of distillate were collected. Concurrently, a control experiment was conducted under identical conditions to ascertain the presence of volatile FA that might be present in the sulfuric acid. The resulting larval distillate was titrated using a 0.1 mol/L potassium hydroxide solution. The amount of volatile FA (in milligrams) in the mealworms was calculated according to the formula suitable for poultry:

(4) $$X=\frac{5.61\times \left(V_{1-}V_{2}\right)\times K\times 100}{g}\;$$

where 5.61—0.1 mol/L titer of potassium hydroxide solution, mg/mL, V1—0.1 mol/L the amount of potassium hydroxide solution used to neutralize the volatile FA in 200 mL of sausages extract, ml, V2—0.1 mol/L amount of potassium hydroxide solution used to neutralize volatile FA in 200 mL control extract, mL, K—correction factor for the molar concentration of potassium hydroxide (1.0–0.1 mol/L for potassium hydroxide solution) and g—mass of the mealworms, g.

### 2.6. Statistics

Statistical analysis was performed utilizing IBM SPSS Statistics 29.0.0.0 (241). The means and standard deviations of the variables under investigation in the different groups were computed. The group differences were assessed through ANOVA with post hoc Bonferroni testing. Statistical significance was determined at a threshold of *p* < 0.05.

## 3. Results and Discussion

### 3.1. Physicochemical Parameters of Sausages

Comparing the control group (SC) with sausages containing lyophilized (SD) and frozen (SF) mealworm larvae showed that the inclusion of mealworms affected various sausage quality parameters, including pH, cooking loss, texture hardness, moisture content, and dry material percentage.

The optimal pH for sausages, based on scientific recommendations, usually falls within a mildly acidic to neutral range, typically around 5.8 to 6.5 [47,48,49]. This range is considered ideal for several reasons: microbial stability, protein functionality, flavor, and color stability [50,51]. Most analyzed samples, including the control (SC) and all SF and SD samples except SD30, had pH values from 6.29 to 6.68, within or near the optimal range, indicating generally favorable qualities based on pH (Table 2). Majcherczyk et al. analyzed the chemical safety and quality attributes of dried sausages and investigated that the pH of the sausage obtained was slightly acidic (pH 6.12 ± 0.007); thus, our results are closer to the neutral medium [52]. The SD30 sample had a noticeably lower pH, which could influence its characteristics, possibly making it more prone to having a firmer texture and potentially offering an additional microbial stability benefit, but it may also impact flavor and color differently from those with a pH closer to the optimal range [53]. Sausages with mealworms showed a slight fluctuation in pH compared to the control, with lyophilized larvae (SD) sausages tending towards a lower pH, indicating a slight increase in acidity, especially noticeable in the SD30 group. Frozen larvae (SF) sausages had a minimal pH increase in SF10 but showed a decrease in SF20 and SF30, aligning with the lyophilized groups in terms of trending towards acidity but being less pronounced. According to Hyun-Wook et al.’s the pH of 10% of untreated mealworm larvae was 6.32 ± 0.08 (in our study, 6.36 ± 0.09), and the control sample was 6.04 ± 0.07, while in our study the pH was much higher—6.61 ± 0.11 [19]. 

Cooking loss in sausages, the percentage of weight loss from water evaporation and fat melting during cooking, is vital for producing high-quality, economically viable, and consumer-acceptable sausages in terms of taste and texture. Cooking loss ranged from 15.14% to 27.37%, with significant differences observed among the groups. Cooking loss decreased in both SF and SD sausages compared to the control, suggesting that mealworm addition could contribute to moisture retention during cooking (*p* < 0.05). The results showed that cooking losses decreased as the percentage of freeze-dried and thermostat-dried larvae in the sausages increased (*p* < 0.05). According to scientists, the amount of protein has a significant influence on this because, analogously, as the number of proteins increases, the water-binding capacity also increases [54]. Although high pH also affects water binding, according to Klettner [55], in our study, researchers Choi et al. presented high boiling losses and high pH values [56]. 

Texture hardness values ranged from 0.1 to 0.4 mJ, with some mealworm-added sausages showing increased hardness, which could be attributed to the protein content and structure of the mealworms. In an investigation, Hyun-Wook et al. pre-treated mealworm larvae in emulsion sausages, and the result showed that, as in our study, mealworm larvae added to sausages had an effect on hardness by increasing it [19]. 

The moisture content in sausages varied with the addition of mealworms, ranging from 36.60% to 50.22%. Sausages with higher percentages of mealworms tended to have higher moisture content, suggesting that mealworms contribute to increased moisture (*p* < 0.05). Interestingly, Hyun-Wook et al. obtained 49.11 ± 0.25% in their study after adding 10% flour larvae. Although the data are similar to our final result, they obtained the opposite trend as the amount of larvae increased and the moisture content decreased. This may have been influenced by the protein content, as their results showed that the larvae contained more protein than the meat [19]. Proteins can act as emulsifiers and hydrocolloids, which help retain water molecules in food products. 

The data suggest that mealworm addition can enhance certain quality attributes like cooking loss and texture while also impacting pH, and moisture content, with specific trends depending on the form and amount of mealworms used.

### 3.2. Nutritional Value

In our study, fat content increased with the percentage of freeze-dried larvae, whereas we did not observe such a trend with dried larvae (*p* < 0.05) (Table 3). Yun-Sang et al. obtained a very similar trend in their study—as the amount of mealworms (not freeze-dried) increased, the amount of fat in sausages decreased [57]. Fat content increased with the inclusion of mealworms, peaking at 28.65 g/100 g in the SD30 sample, suggesting that mealworm addition can significantly alter the fat profile of sausages. In our previous study, while analyzing the influence of different substrates on mealworms, we noticed that when grown on brewers’ spent grain, the fat content was 20.23%, while when it was added to sausages, the fat content increased proportionally as the concentration of mealworms increased, so the highest fat content was found in sausages with 30% lyophilized mealworms (28.65%) [14]. 

The data indicated a decrease in cholesterol levels in sausages with the inclusion of mealworms, both lyophilized and dried, compared to the control. The highest reduction was observed in sausages with a 30% addition of both types of mealworms, suggesting that incorporating mealworms can significantly lower the cholesterol content in sausages, potentially making them a healthier option. Emel Cengiz conducted a study on frankfurter-type sausages, altering cholesterol levels through fat reduction and fat replacer addition. Results revealed that reducing fat levels from 20% to 10% and 5% led to decreases in cholesterol contents of 32.0% and 45.8%, respectively [58]. 

Hydroxyproline, a key collagen component, is vital for its stability and influences the structural integrity and texture of meat products, affecting their gelation properties, water-binding capacity, and juiciness during thermal processing [59]. The control sample showed a moderate level of hydroxyproline, suggesting a standard collagen content. Inclusion of mealworms tended to alter hydroxyproline levels, with a noticeable decrease in sausages containing higher percentages of mealworms. 

In sausages, collagen enhances texture, elasticity, and juiciness, improves structure and water retention for maintained juiciness during cooking, and transforms into gelatin, influencing flavor and nutritional value [55,56,57,58,59,60,61,62]. In our study, the collagen content decreased with the addition of 10% and 20% lyophilized mealworms compared to the control sample. However, sausages with dried mealworms showed an increase in collagen content at 10% inclusion, then a decrease as the proportion of mealworms increased to 20% and 30%. This suggests that the form of mealworms (lyophilized vs. dried) and their proportion in sausages significantly influence collagen levels, potentially affecting texture.

Data shows that incorporating mealworms, both lyophilized and dried, into sausages increases salt content, particularly with dried mealworms, potentially affecting flavor and preservation and necessitating seasoning adjustments for taste and stability. The inclusion of mealworms in both lyophilized and dried forms at varying concentrations significantly influences the nutritional and textural properties of sausages. Particularly, the addition of mealworms increases fat content, especially with lyophilized forms at higher concentrations, and modifications in the sausage’s structural integrity through changes in collagen content. These findings underscore the impact of mealworm incorporation on enhancing nutritional value, such as altering FA profiles, and on the physical properties of sausages, suggesting a need for tailored adjustments in formulations to optimize both health benefits and sensory attributes.

#### 3.2.1. Amino Acid Content

Essential amino acids are vital for protein synthesis, supporting the growth, repair, and maintenance of body tissues [63,64]. They also play a crucial role in various metabolic and physiological processes, including enzyme and hormone production, immune function, and nutrient absorption [65]. 

For most amino acids listed, the content varied between the control group (SC) and those with added mealworms (SF and SD series), indicating that mealworms either contribute directly to the amino acid content or influence the overall amino acid profile through their interaction with the meat matrix (Table 4). Aspartic acid and glutamic acid are essential for neurotransmission [66,67], metabolism, immune support, detoxification, and heart health, and are used as flavor enhancers in foods [68,69]. Samples with lyophilized mealworms (SD series) generally showed higher levels of aspartic acid and glutamic acid compared to the control and those with dried mealworms. In our previous study, specifically in a sample labeled 3L, we found that lyophilized mealworms grown on brewers’ spent grain had aspartic acid 4.30 ± 0.257 and glutamic acid 6.49 ± 0.903 mg/kg. This explains why sausages with freeze-dried larvae contained statistically more of these amino acids (*p* < 0.05) [70]. Proline, involved in collagen stability, plays an important role in protein synthesis and structure, nutrition, wound healing, antioxidative reactions, and immune responses [71]. The increased content of these amino acid levels was in the SD10 sample, suggesting potential differences in connective tissue content or structure among the samples. Isoleucine and leucine support protein synthesis and muscle repair [71], threonine aids immune function [72], lysine assists in hormone and enzyme production, and phenylalanine is crucial for amino acid biosynthesis and neurotransmitter production, collectively impacting mental health, muscle growth, and immune response [73,74]. A statistically higher amount of essential FA phenylalanine, isoleucine, leucine, threonine, and lysine was determined in the SD10 sample (*p* < 0.05), while the valine content only in the SD20 sample (0.42 ± 0.124) exceeded the concentration of the latter (0.40 ± 0.085). Therefore, of the six essential FAs identified, four had the highest amount after adding 10% freeze-dried mealworms. 

The summary of the total amino acids indicates a decrease in the total amino acid content as the proportion of mealworms increases, with the lowest totals observed in the SF30 and SD30 samples, except for SF10. This suggests that while mealworms can be a source of proteins and specific amino acids, their inclusion at higher percentages may dilute the overall concentration of essential amino acids, possibly affecting the nutritional quality of the sausages.

#### 3.2.2. FA Content

Expanding on our earlier publication by Jankauskienė et al., we intentionally selected larvae labeled as 3L samples that were raised on a brewer’s spent grain substrate for sausage production. These larvae exhibited the most favorable FA composition, particularly in terms of oleic acid content, compared to other rearing conditions [70]. 

The addition of freeze-dried larvae increased the amount of unsaturated C18:1 FA and omega-6 (C18:2 w6) FA (Table 5). It was also observed that both processing methods decreased the content of certain saturated fatty acids (SFAs), such as C10:0, C14:0, and C12:0, indicating the complex effect of these ingredients on the FA profile of sausages; however, no significant difference was observed. SFAs are linked to cardiovascular health risks, but the impact varies by specific FA types [75]. Dietary intervention continues to be the primary choice for the prevention and treatment of cardiovascular diseases by modulating the intake of SFAs in the diet [76]. The levels of Long-chain (C14:0, C16:0, C18:0) SFAs show variability with mealworm inclusion, with some samples like SD30 showing a substantial increase in certain SFA acids (C14:0). The research outlined by Gillingham et al. in their work reveals that monounsaturated fatty acids (MUFAs) contribute to the reduction in detrimental cholesterol and may also elevate the levels of protective cholesterol [77]. Estruch et al.’s research confirms that a Mediterranean diet high in MUFAs from olive oil and nuts reduces major cardiovascular events, highlighting the benefits of MUFAs over SFAs [78]. Monounsaturated fats can help reduce increased cholesterol levels and are a healthier fat choice. MUFAs (C16:1, C17:1, C18:1), known for their heart-healthy properties, are maintained or slightly increased in mealworm-added sausages, but there was no statistically significant difference between frozen and lyophilized larvae, and no specific trends were detected.

PUFAs, particularly omega-3 and omega-6 fatty acids, are vital for human and animal health due to their importance in cell membrane structure, anti-inflammatory properties [79,80], and role in cardiovascular health and disease prevention, as highlighted by Simopoulos and Calder’s research [76,77,81,82]. PUFAs such as C18:2 ω6, C18:3 α ω3, C20:4 ω6 show a general increase, particularly in sausages with higher mealworm content (e.g., SF30, SD30) (*p* < 0.05). The PUFA content progressively increased with higher mealworm incorporation. The highest PUFA content was in SD30 (lyophilized mealworms at 30% inclusion).

Extensive research shows that omega-3 fatty acids offer various health benefits [83], including reducing the risk of cardiovascular diseases like coronary heart disease and myocardial infarction and potentially lowering the risk of cancer, Alzheimer’s disease, dementia, and age-related macular degeneration [81,84,85,86]. The incorporation of mealworm larvae into sausages led to several changes in the omega-3 FA composition (C18:3 α ω3, C22-5 ω3, C22-6 ω3). The inclusion of lyophilized mealworm larvae (SD), particularly at a 30% level (SD30), increased the amount of alpha-linolenic acid (C18:3 α w3) to 1.05 ± 0.012, compared to the control sample (0.90 ± 0.004), and the quantity of C20:3 w3 escalated from 0.11 ± 0.007 in the control sample to 0.59 ± 0.319 in the SD30 sample, indicating a potential improvement in the omega-3 FA profile with a statistically increasing quantity of lyophilized larvae (*p* < 0.05). Omega-6 fatty acid content also showed a rising trend with increased mealworm content, peaking at 18.13% in SD30. This increment is statistically significant, demonstrating the impact of higher mealworm inclusions on omega-6 fatty acid levels (*p* < 0.05). The Omega 6/3 ratio exhibited a decreasing trend as the inclusion of mealworms increased, especially notable in SF30, which had the lowest ratio of 6.10. This ratio indicates a more balanced fatty acid profile in sausages with a higher mealworm content. The statistical data (ranging from a high of 10.00 in SF20 to a low of 6.10 in SF30) further confirm that mealworm inclusion impacts the omega fatty acid balance.

In conclusion, the incorporation of mealworms into sausage formulations appears to influence the FA composition, with a shift towards a potentially more beneficial profile, including higher levels of monounsaturated and PUFAs, especially omega-3s. This shift could enhance the nutritional value of sausages, offering health benefits such as improved cardiovascular health and cognitive function. However, the increased levels of certain saturated fats in some samples suggest the need for a balanced approach to mealworm inclusion to maximize health benefits while minimizing risks.

### 3.3. Safety Parameters of Sausages

#### 3.3.1. Biogenic Amines

Biogenic amines, organic nitrogen compounds formed through amino acid decarboxylation or amination facilitated by bacterial enzymes [87,88], are closely linked to microbial activity in food products like sausages [89]. This activity can be influenced by factors like the composition of the food, the storage conditions, and the presence of microorganisms capable of producing decarboxylase enzymes [90]. It is important to note that the larvae, like the meat for the sausages, were kept under similar controlled conditions, so the influence of the storage conditions should be ruled out as the cause of the formation. Biogenic amines, though essential for normal physiological functions like histamine’s role in gastric acid secretion and immune responses [91], can pose health risks when consumed excessively, leading to symptoms of food poisoning such as headaches [92,93], flushing, and hypotension. Thus, monitoring and regulating biogenic amine levels in food products, particularly fermented foods, are critical for ensuring food safety and public health [94,95]. 

The control sample (SC) consisted of basic ingredients without mealworms and showed negligible or undetectable levels of most amines, except for Spermine and Spermidine, which were present in significant amounts (Table 6). However, samples with mealworms (both lyophilized and dried, at various percentages) showed increased levels of certain amines, indicating that the inclusion of mealworms either introduces new sources of amino acids for decarboxylation or affects the microbial flora in a way that promotes the production of these amines. While there is no specific legal stipulation for the permissible histamine levels in edible insects, drawing parallels from the standards set for fishery products, the histamine content should not surpass 200 mg/kg as outlined by Regulation (EC) No. 2073/2005 [96]. Our study has shown that the amount of histamine did not exceed the limit of detection in all samples. In the documentation provided by scientists at the EFSA, the concentration of cadaverine found in larvae was reported to fall within the range of 6.66 to 8.01 milligrams per kilogram. In our study, after inserting mealworms into sausages, the amounts varied from 4.60 ± 0.12 with lyophilized larvae to 4.60 ± 0.12 with frozen [97]. The detected levels of cadaverine in larvae were relatively low when compared to their levels in other food items. For context, cadaverine can reach much higher concentrations in certain foods, such as up to 3170 milligrams per kilogram in cheeses, up to 1690 milligrams per kilogram in fish and fish products, and notably in fermented sausages, among others [98,99]. The data underscored the statistical significance (*p* < 0.05) of variations among the groups, demonstrating the influence of mealworm inclusion on sausage safety parameters, with an increase in mealworm content being directly associated with higher levels of certain amines, such as cadaverine, putrescine, tyramine, and spermidine, compared to the control group. The amount of cadaverine and tyramine was found to be statistically lower in sausages containing lyophilized larvae compared to frozen ones (*p* < 0.05). According to the European Food Safety Authority (EFSA), putrescine and cadaverine are identified as the most frequently occurring biogenic amines in various food products [97]. However, the opposite trend was found with putrescine and spermine—a higher amount of this substance was detected in sausages with dried larvae, but no significant difference was found. 

Nelly C. Muñoz Esparza and her team found that the levels of spermine in beef, pork, and chicken exceeded 148 nmol/g [100]. This was consistent across both fresh and processed versions of these meats, showing no significant differences in their spermine content. The lyophilization process may alter the availability of free amino acids, which are precursors to biogenic amine formation. The freezing and drying phases could modify the protein structures in food, potentially making amino acids more accessible to decarboxylase enzymes, which could either increase or decrease the formation of biogenic amines depending on the specific conditions and the presence of active enzymes [101,102]. The discrepancy in cadaverine levels between sausages prepared with frozen and lyophilized larvae could stem from the more effective reduction in microbial activity and enzyme deactivation achieved through lyophilization compared to freezing. This highlights the importance of the preservation method chosen for controlling the formation of biogenic amines in food products [103]. Spermine’s antioxidant properties may reduce the risk of chronic diseases like cancer and cardiovascular disease by protecting cells from free radical damage [104], while also playing a role in cellular growth regulation and immune response modulation, emphasizing its significance in maintaining cellular health and preventing disease [105]. Elena Bartkiene et al. have studied the formation of biogenic amines in baked bread with cricket flour and have found that the amount of spermine was also formed in cricket flour, as much as 307.2 ± 21.84 mg/kg, while our study showed that the amount of spermine decreased by adding a higher percentage of mealworms compared to the control group of pork sausages [106]. In all samples, except for spermine, the content of cadaverine, putrescine, tyramine, and spermidine increased proportionally with the concentration of mealworms (*p* < 0.05). In a previously published article, we identified notably elevated levels of biogenic amines in mealworm larvae, hypothesizing a potential influence from microorganisms residing within the larvae’s bodies [41]. 

Thus, in summary, it can be concluded that the amount of biogenic amines formed after the introduction of mealworms into sausages increased in all cases, except for spermine, but this does not have a significant impact on the overall safety of sausages. But, in any case, further research is essential to understand the factors influencing biogenic amine production in mealworm-incorporated sausages, aiming to develop methods for mitigating these levels in extensive production settings while ensuring consistent raw material and process conditions to keep amine concentrations within safe limits. Additionally, effective monitoring and control strategies need to be implemented to prevent unacceptable increases in biogenic amines, which involves optimizing fermentation conditions, storage, and handling procedures to reduce amine formation.

#### 3.3.2. Volatile FA

**Figure 1:**
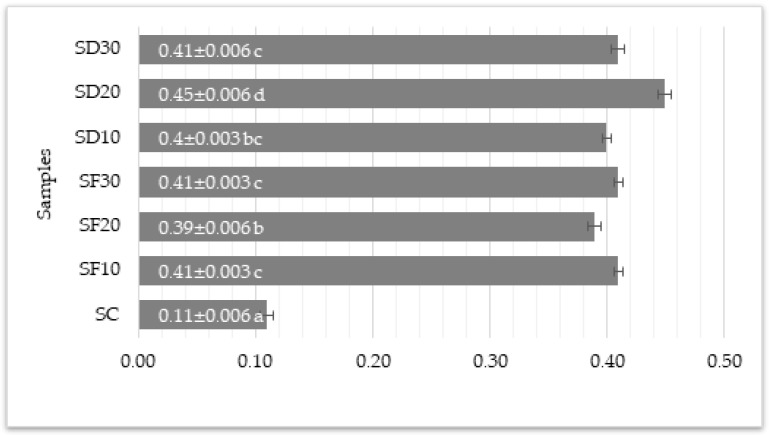
Volatile FA content, average ± standard deviation, n = 3. a,b,c,d—means marked with different letters differed statistically significantly (*p* < 0.05, Bonferroni criterion); SC—control sample (lean pork + back fat + ice + salt + pepper); SD10—sausages with lyophilized mealworms (lean pork + back fat + 10% mealworms + ice + salt + pepper); SD20—sausages with lyophilized mealworms (lean pork + back fat + 20% mealworms + ice + salt + pepper); SD30—sausages with lyophilized mealworms (lean pork + back fat + 30% mealworms + ice + salt + pepper); SF10—sausages with dried mealworms (lean pork + back fat + 10% mealworms + ice + salt + pepper); SF20—sausages with dried mealworms (lean pork + back fat + 20% mealworms + ice + salt + pepper); SF30—sausages with dried mealworms (lean pork + back fat + 30% mealworms + ice + salt + pepper).

According to the data from our research, a significant increase in volatile fatty acid levels in all sausage samples with added mealworms compared to the control sample was indicated (Figure 1). The control sample showed the lowest levels of volatile fatty acids (0.11 ± 0.010 meq/kg of fat), suggesting it is the freshest or the least degraded among the samples tested. In contrast, the samples with mealworms exhibited considerably higher levels, ranging from 0.39 to 0.46 meq/kg of fat, indicating that the addition of mealworms, whether lyophilized or dried, affected the volatile fatty acid content in sausages. The highest levels observed in the SD20 samples (0.45 ± 0.010 meq/kg of fat) suggested that the proportion of mealworms might also play a role in the degree of fatty acid volatility, potentially indicating a higher rate of decomposition or different spoilage dynamics in those samples. The concentration of volatile fatty acids was measured following the guidelines set forth by the Minister of Agriculture of the Republic of Lithuania, which stipulate that for the evaluation of meat and poultry freshness, the level of volatile fatty acids in mealworms must not surpass 0.35 mL [107]. However, none of the tested samples containing mealworms exceeded the recommended limit. This limitation may be attributed to the distinct chemical compositions observed between brewers’ spent grain and alternative growth mediums, potentially affecting the chemical reactions and metabolic processes within the larvae [14]. From our earlier research, it was observed that brewers’ spent grain contains a notably higher content of fats and oils, which are prone to initiating oxidation when exposed to air or other oxidizing agents [14]. This increase could be attributed to several factors, including the natural composition of the mealworms, the interaction between mealworm components and the meat matrix during storage and processing, or a faster rate of lipid oxidation or microbial action in the mealworm-containing sausages. 

#### 3.3.3. Peroxide Value

Peroxide values are a key indicator of lipid oxidation in foods, reflecting the initial stages of fat rancidity, which can affect the flavor, nutritional value, and safety of the product [108,109]. 

**Figure 2:**
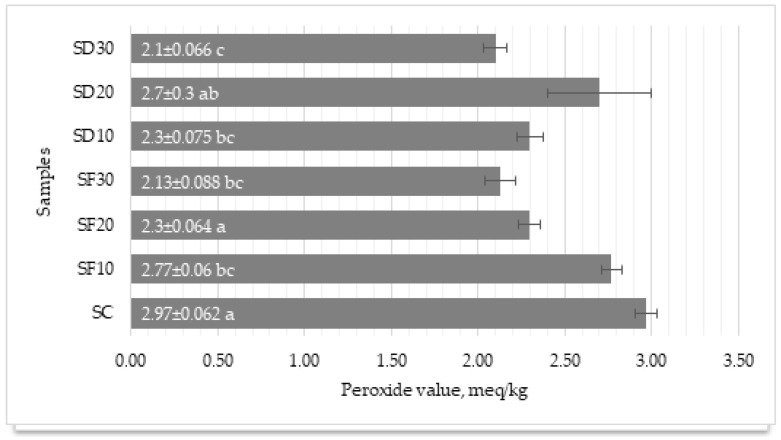
Peroxide value, average ± standard deviation, *n* = 3. a,b,c—means marked with different letters differed statistically significantly (*p* < 0.05, Bonferroni criterion); SC—control sample (lean pork + back fat + ice + salt + pepper); SC—control sample (lean pork + back fat + ice + salt + pepper); SD10—sausages with lyophilized mealworms (lean pork + back fat + 10% mealworms + ice + salt + pepper); SD20—sausages with lyophilized mealworms (lean pork + back fat + 20% mealworms + ice + salt + pepper); SD30—sausages with lyophilized mealworms (lean pork + back fat + 30% mealworms + ice + salt + pepper); SF10—sausages with dried mealworms (lean pork + back fat + 10% mealworms + ice + salt + pepper); SF20—sausages with dried mealworms (lean pork + back fat + 20% mealworms + ice + salt + pepper); SF30—sausages with dried mealworms (lean pork + back fat + 30% mealworms + ice + salt + pepper).

In our research, the control sample (SC) showed a peroxide value of 2.97 meqv/kg of fat, which was within a narrow range, indicating stable lipid quality with minimal oxidation (Figure 2). The samples with added mealworms (both SF and SD series) generally exhibited a range of peroxide values, with most showing lower values compared to the control, suggesting reduced levels of lipid oxidation. Notably, the SF30 and SD30 samples showed the lowest peroxide values (2.13 ± 0.152 and 2.1 ± 0.115, respectively), implying that higher concentrations of mealworms might contribute to lower lipid oxidation rates compared to the control group (*p* < 0.005). This finding could suggest several possibilities, such as that mealworms might possess natural antioxidants that help protect the lipids in the sausages from oxidation. Minhee Baek et al. investigated the processing effects on the antioxidant activity of mealworm larvae. Their assays confirmed that ABTS-only freeze-dried mealworms exhibited higher activity [110]. The process of lyophilization or drying of mealworms, as well as their integration into the sausage matrix, could influence the overall oxidative stability of the fats within the sausages. Compared to the regulations set forth in ISO 27107:2010, which apply to both animal and vegetable fats and oils, it is recommended to consider fats as oxidized if their peroxide content surpasses 10 meq/kg, so our results were almost five times lower [111]. According to Ketinun Kittipongpittaya, normally fresh, quality pork should have a low peroxide value, showing minimal oxidation of up to 5 meq O^2^ kg fat already on the first day, which is a higher value than found in this study [112]. 

Kröncke et al. study results have shown that freeze-drying mealworms leads to significantly higher oxidation compared to other drying methods [113]. Contrarily, our findings did not show a statistical difference; however, sausages containing dried larvae exhibited a higher peroxide level than those with lyophilized larvae. 

In conclusion, the inclusion of mealworms in sausage formulations appears to have a beneficial effect on lipid oxidative stability, as indicated by generally lower peroxide values. This effect is particularly evident with higher mealworm inclusion rates, suggesting potential antioxidant effects or beneficial changes in lipid composition.

#### 3.3.4. Content of Nitrate and Nitrite 

Nitrates and nitrites form in sausages naturally or are intentionally added as curing agents [114], with the latter used to inhibit pathogenic microorganisms like Clostridium botulinum, enhance flavor, and stabilize the pink color of cured meats [115]. In all sausage samples, including those with added mealworms (both lyophilized and dried) and the control sample, the nitrate and nitrite levels were found to be below 2 mg/kg and significantly felt within the safe consumption limits as per scientific and regulatory standards (Appendix A). The mealworms themselves showed a nitrate level of 5 ± 1.00 mg/kg, which still represented a low risk, especially when incorporated into the sausages in small percentages, as evidenced by the resultant sausage nitrate and nitrite concentrations remaining low. According to the European Food Safety Authority (EFSA), the acceptable daily intake (ADI) for nitrates is 3.7 milligrams per kilogram of body weight per day (mg/kg bw/day), and for nitrites, it is set at a more conservative level close to 0.07 mg/kg bw/day [116]. The low levels of nitrates and nitrites in all the sausage samples indicated that their consumption posed minimal risk concerning these compounds, aligning with EFSA guidelines and reaffirming their safety from a nitrate and nitrite perspective. Such a low level suggested that the inclusion of mealworms, whether lyophilized or dried, did not significantly alter the safety parameters of the sausages in terms of nitrate and nitrite content, making them suitable for consumption under current food safety guidelines.

## 4. Conclusions

The inclusion of mealworms in pork sausage formulations has been shown to increase fat content, particularly when using freeze-dried mealworms. Moreover, this study highlights the potential health benefits of incorporating mealworms into sausages, such as reduced cholesterol levels and altered collagen content, which could have implications for the product’s texture and nutritional value. The nutritional profile showed varying impacts; however, larvae subjected to lyophilization demonstrated a superior influence on the nutritional value compared to larvae subjected to drying. Upon comparing the control sample with sausages enriched with mealworms, an augmentation in fatty acids, specifically MUFAs and PUFAs, including an emphasis on omega-3 fatty acids, was observed. The inclusion of *T. monitor* in sausages had a positive effect on the quality of amino acids but a negative effect on the quantity, and the best results were obtained with 10% of lyophilized mealworms. Although almost all biogenic amines increased with the increasing percentage of mealworms in sausages, it is crucial to note that they did not exceed the regulatory limits set for other food products. Additionally, the levels observed were significantly lower. Therefore, adding mealworms to sausages is not expected to have a significant impact on the overall safety results. The content of nitrates and nitrites in the larvae was extremely low, so their use as an ingredient in sausages did not affect the safety parameters and was not statistically different from the control group. The integration of mealworms into sausages has also reduced the value of peroxides due to the possible presence of natural antioxidants and thus improved the safety parameters of the sausages. Therefore, future research should delve into the long-term health impacts of consuming mealworm-enhanced sausages, particularly their effects on cholesterol, heart health, and allergenic potential, to better understand the outcomes of regular intake. Investigations into the optimal mix of lyophilized versus dried mealworms could refine sausage formulations to enhance their nutritional profile, taste, and texture, tailored through the diet and breed of the mealworms used. Additionally, expanding the scope to include comparative analyses of various edible insects, alongside assessments of consumer acceptance and the nutritional benefits of natural antioxidants in mealworms, could increase the development of safe, environmentally sustainable, and economically viable insect-based food products.

## Figures and Tables

**Table 1:** Sausage formulations for replacing pork meat with mealworms.

Ingredient/Group	Control Sample	Frozen and Freeze-Drying Mealworms			Frozen and Dried Mealworms (in Thermostat)		
	SC	SD10	SD20	SD30	SF10	SF20	SF30
Lean pork ^1^	55	45	35	25	45	35	25
*T. monitor* larvae ^1^	0	10	20	30	10	20	30
Back fat ^1^	25	25	25	25	25	25	25
Ice ^1^	20	20	20	20	20	20	20
Salt ^2^	1.5	1.5	1.5	1.5	1.5	1.5	1.5
Pepper ^2^	1.5	1.5	1.5	1.5	1.5	1.5	1.5

^1^ Ingredients in %; ^2^ ingredients in g.

**Table 2:** Physicochemical and textural properties of sausages with lyophilized and dried mealworms, average ± standard error, *n* = 3.

	pH	Cooking Loss, %	Texture Hardness, mJ	Moisture Content, %
SC	6.61 ± 0.11 a	25.94 ± 0.19 a	0.3 ± 0.020 a	42.69 ± 0.21 a
SF10	6.68 ± 0.01 a	27.37 ± 0.28 a	0.4 ± 0.050 b	36.60 ± 0.15
SF20	6.51 ± 0.02 a	21.68 ± 0.64 b	0.2 ± 0.001 c	46.42 ± 0.10 d
SF30	6.51 ± 0.08 a	16.98 ± 0.91 c	0.1 ± 0.010 d	48.07 ± 0.22 c
SD10	6.36 ± 0.09 a	21.69 ± 0.43 b	0.2 ± 0.003 c	44.59 ± 0.19 f
SD20	6.40 ± 0.12 a	19.84 ± 0.28 d	0.3 ± 0.010 a	50.00 ± 0.12 e
SD30	6.29 ± 0.82 a	15.14 ± 0.92 e	0.2 ± 0.005 c	50.22 ± 0.26 e

a,b,c,d,e,f—means marked with different letters in the column differed statistically significantly (*p* < 0.05, Bonferroni criterion); SC—control sample (lean pork + back fat + ice + salt + pepper); SD10—sausages with lyophilized mealworms (lean pork + back fat + 10% mealworms + ice + salt + pepper); SD20—sausages with lyophilized mealworms (lean pork + back fat + 20% mealworms + ice + salt + pepper); SD30—sausages with lyophilized mealworms (lean pork + back fat + 30% mealworms + ice + salt + pepper); SF10—sausages with dried mealworms (lean pork + back fat + 10% mealworms + ice + salt + pepper); SF20—sausages with dried mealworms (lean pork + back fat + 20% mealworms + ice + salt + pepper); SF30—sausages with dried mealworms (lean pork + back fat + 30% mealworms + ice + salt + pepper).

**Table 3:** Chemical composition of sausages formulated with lyophilized and dried mealworm larvae, average ± standard error, *n* = 3.

	Fat, g/100 g	Ash, %	Cholesterol, mg/100 g	Hydroxyproline, g/100 g	Salt Content, %	Collagen, g/100 g
SC	23.20 ± 0.11 a	0.97 ± 0.021 a	73.86 ± 1.14 a	0.23 ± 0.010 a	1.48 ± 0.01 a	1.84 ± 0.08 a
SF10	26.55 ± 0.14 b	0.98 ± 0.002 a	64.08 ± 1.65 b	0.19 ± 0.010 bc	2.32 ± 0.10 b	1.52 ± 0.08 b
SF20	26.50 ± 0.03 b	0.98 ± 0.160 a	61.74 ± 1.25 b	0.17 ± 0.010 bd	4.06 ± 0.07 c	1.36 ± 0.08 bc
SF30	25.59 ± 0.06 c	0.98 ± 0.031 a	56.40 ± 0.98 c	0.21 ± 0.012 ac	5.8 ± 0.08 d	1.71 ± 0.09 ab
SD10	24.27 ± 0.04 d	0.98 ± 0.002 a	64.36 ± 2.11 b	0.27 ± 0.010 e	4.64 ± 0.18 e	2.16 ± 0.08 d
SD20	24.50 ± 0.04 ef	0.98 ± 0.008 a	55.96 ± 1.42 c	0.16 ± 0.006 d	5.22 ± 0.09 f	1.25 ± 0.05 c
SD30	28.65 ± 0.05	0.99 ± 0.001 a	55.65 ± 1.53 c	0.15 ± 0.010 d	6.38 ± 0.3 g	1.20 ± 0.08 c

a,b,c,d,e,f,g—means marked with different letters in the column differed statistically significantly (*p* < 0.05, Bonferroni criterion); SC—control sample (lean pork + back fat + ice + salt + pepper); SD10—sausages with lyophilized mealworms (lean pork + back fat + 10% mealworms + ice + salt + pepper); SD20—sausages with lyophilized mealworms (lean pork + back fat + 20% mealworms + ice + salt + pepper); SD30—sausages with lyophilized mealworms (lean pork + back fat + 30% mealworms + ice + salt + pepper); SF10—sausages with dried mealworms (lean pork + back fat + 10% mealworms + ice + salt + pepper); SF20—sausages with dried mealworms (lean pork + back fat + 20% meal-worms + ice + salt + pepper); SF30—sausages with dried mealworms (lean pork + back fat + 30% mealworms + ice + salt + pepper).

**Table 4:** Amino acid composition of sausages formulated with lyophilized and dried mealworm larvae, g/100 g, average ± standard error, *n* = 3.

	SC	SF10	SF20	SF30	SD10	SD20	SD30
Aspartic Acid	1.64 ± 0.029 a	1.31 ± 0.130 b	1.09 ± 0.087 bc	0.95 ± 0.032 c	2.21 ± 0.121 d	1.74 ± 0.069 a	1.00 ± 0.037 c
Glutamic acid	2.04 ± 0.039 a	1.65 ± 0.059 b	1.32 ± 0.110 c	1.12 ± 0.022 c	2.71 ± 0.133 d	2.10 ± 0.075 a	1.19 ± 0.029 c
Asparagine	n.d.	n.d.	n.d.	n.d.	n.d.	n.d.	n.d.
Serine	0.55 ± 0.021 ba	0.49 ± 0.036 b	0.41 ± 0.044 cb	0.37 ± 0.003 c	0.79 ± 0.034 d	0.63 ± 0.022 a	0.39 ± 0.008 c
Glycine	n.d.	n.d.	n.d.	n.d.	1.00 ± 0.192	0.80 ± 0.046	n.d.
Threonine	0.70 ± 0.049 ab	0.64 ± 0.036 bc	0.51 ± 0.043 cd	0.43 ± 0.010 d	1.05 ± 0.061 e	0.81 ± 0.078 a	0.46 ± 0.020 d
Arginine	0.81 ± 0.012 ac	0.65 ± 0.047 a	0.48 ± 0.014 b	0.41 ± 0.023 b	0.92 ± 0.125 c	0.80 ± 0.047 ac	0.42 ± 0.021 b
Alanine	0.72 ± 0.020 a	0.66 ± 0.031 a	0.56 ± 0.021 b	0.54 ± 0.015 b	1.07 ± 0.034 c	0.89 ± 0.040 d	0.56 ± 0.016 b
Tyrosine	0.40 ± 0.014 a	0.38 ± 0.056 a	0.31 ± 0.024 a	0.32 ± 0.021 a	0.59 ± 0.054 b	0.55 ± 0.050 b	0.37 ± 0.025 a
Cystine	1.46 ± 0.116 ab	1.07 ± 0.099 ab	0.88 ± 0.083 a	0.93 ± 0.055 a	1.16 ± 0.419 ab	1.62 ± 0.280 b	0.93 ± 0.145 a
Valine	0.40 ± 0.053 ab	0.28 ± 0.063 ab	0.19 ± 0.004 a	0.19 ± 0.043 a	0.40 ± 0.085	0.42 ± 0.124 c	0.22 ± 0.045
Methionine	n.d.	n.d.	n.d.	n.d.	n.d.	n.d.	n.d.
Tryptophan	0.16 ± 0.017 ab	0.13 ± 0.026 bc	0.08 ± 0.011 c	0.08 ± 0.017 c	0.20 ± 0.027 bc	0.16 ± 0.037 ab	0.08 ± 0.025 ab
Phenylalanine	0.59 ± 0.020 a	0.49 ± 0.028 b	0.39 ± 0.042 c	0.34 ± 0.007 c	0.76 ± 0.060 d	0.61 ± 0.015 a	0.35 ± 0.023 c
Isoleucine	0.43 ± 0.006 a	0.34 ± 0.017 b	0.27 ± 0.021 bc	0.24 ± 0.016 c	0.59 ± 0.061 d	0.47 ± 0.027 a	0.26 ± 0.018 bc
Leucine	1.02 ± 0.032 a	0.82 ± 0.046 b	0.66 ± 0.073 bc	0.58 ± 0.018 c	1.36 ± 0.117 d	1.06 ± 0.031 a	0.61 ± 0.031 c
Lysine	0.93 ± 0.014 a	0.72 ± 0.046 ab	0.44 ± 0.131 c	0.44 ± 0.016 c	1.19 ± 0.133 d	0.93 ± 0.012 a	0.48 ± 0.034 c
Proline	1.04 ± 0.107 ab	1.00 ± 0.215 ab	0.71 ± 0.152 a	1.11 ± 0.209 ab	1.95 ± 0.312 c	1.48 ± 0.078 bc	0.71 ± 0.143 a

a,b,c,d,e—means marked with different letters in the row differed statistically significantly (*p* < 0.05, Bonferroni criterion); SC—control sample (lean pork + back fat + ice + salt + pepper); SD10—sausages with lyophilized mealworms (lean pork + back fat + 10% mealworms + ice + salt + pepper); SD20—sausages with lyophilized mealworms (lean pork + back fat + 20% mealworms + ice + salt + pepper); SD30—sausages with lyophilized mealworms (lean pork + back fat + 30% mealworms + ice + salt + pepper); SF10—sausages with dried mealworms (lean pork + back fat + 10% mealworms + ice + salt + pepper); SF20—sausages with dried mealworms (lean pork + back fat + 20% mealworms + ice + salt + pepper); SF30—sausages with dried mealworms (lean pork + back fat + 30% mealworms + ice + salt + pepper).

**Table 5:** FA composition of sausages with lyophilized and dried mealworm larvae, average ± standard error, *n* = 3.

	SC	SF10	SF20	SF30	SD10	SD20	SD30
C10:0	0.05 ± 0.003 a	0.03 ± 0.025 a	0	0	0.02 ± 0.038 a	0.02 ± 0.019 a	0.04 ± 0.061 a
C12:0	0.06 ± 0.008 a	0.07 ± 0.026 a	0	0.12 ± 0.207 a	0.08 ± 0.036 a	0.06 ± 0.018 a	0.71 ± 1.036 a
C14:0	1.14 ± 0.014 a	1.17 ± 0.025 a	0.92 ± 0.232 a	1.42 ± 0.239 a	1.20 ± 0.048 a	1.25 ± 0.047 a	1.69 ± 0.684 a
C15:0	0.06 ± 0.006 a	0.06 ± 0.005 a	0.02 ± 0.026 a	0.03 ± 0.023 a	0.05 ± 0.010 a	0.05 ± 0.016 a	0.04 ± 0.007 a
C16:0	22.23 ± 0.281 a	22.45 ± 0.053 a	22.22 ± 0.326 a	21.98 ± 0.710 a	22.06 ± 0.030 a	22.15 ± 0.174 a	21.94 ± 0.191 a
C16:1	1.63 ± 0.009 a	1.61 ± 0.016 a	1.50 ± 0.112 a	1.52 ± 0.096 a	1.62 ± 0.031 a	1.60 ± 0.035 a	1.48 ± 0.091 a
C17:0	0.44 ± 0.013 a	0.44 ± 0.009 ab	0.32 ± 0.069 b	0.34 ± 0.043 ab	0.42 ± 0.018 ab	0.40 ± 0.031 ab	0.33 ± 0.052 ab
C17:1	0.36 ± 0.012 a	0.37 ± 0.009 a	0.26 ± 0.085 a	0.28 ± 0.041 a	0.35 ± 0.012 a	0.34 ± 0.019 a	0.28 ± 0.074 a
C18:0	13.99 ± 0.057 a	13.26 ± 0.114 d	12.69 ± 0.206 bc	12.22 ± 0.352 be	12.98 ± 0.051 cd	12.44 ± 0.030 be	12.03 ± 0.020 e
C18:1 tr.	0.18 ± 0.014 a	0.16 ± 0.016 a	0.05 ± 0.066 b	0.08 ± 0.031 ab	0.14 ± 0.022 ab	0.14 ± 0.027 ab	0.10 ± 0.031 ab
C18:1	41.56 ± 0.018 a	42.10 ± 0.101 ac	43.68 ± 0.710 b	43.02 ± 0.464 bc	42.55 ± 0.198 abc	42.52 ± 0.298 abc	42.16 ± 0.840 ac
C18:2 w6	15.22 ± 0.239 a	15.48 ± 0.028 ab	16.26 ± 0.606 ab	16.19 ± 0.574 ab	15.69 ± 0.068 ab	16.33 ± 0.249 ab	16.70 ± 0.697 b
C20:0	0.18 ± 0.014 a	0.15 ± 0.026 ab	0.03 ± 0.044 a	0.03 ± 0.043 a	0.13 ± 0.036 ab	0.12 ± 0.058 ab	0.08 ± 0.079 ab
C18:3 α w3	0.90 ± 0.004 a	0.88 ± 0.010 a	0.65 ± 0.196 a	1.05 ± 0.012 a	0.88 ± 0.016 a	0.88 ± 0.010 a	0.76 ± 0.129 a
C20:1	0.74 ± 0.030 a	0.69 ± 0.039 a	0.33 ± 0.305 a	0.64 ± 0.120 a	0.68 ± 0.015 a	0.63 ± 0.034 a	0.48 ± 0.129 a
C18:3 ґ w6	0.03 ± 0.010 a	0.01 ± 0.020 a	0	0.01 ± 0.415 a	0.01 ± 0.017 a	0.01 ± 0.034 a	0.76 ± 0.134 a
C21:0	0.04 ± 0.004 a	0.02 ± 0.028 a	0.21 ± 0.367 a	0	0	0.01 ± 0.021 a	0
C20:2 w6	0.62 ± 0.005 a	0.58 ± 0.016 ac	0.38 ± 0.119	0.42 ± 0.057 bc	0.57 ± 0.028 abc	0.52 ± 0.039 abc	0.42 ± 0.092 b
C22:0	0.01 ± 0.012	0	0	0	0	0	0
C20:3 w6	0.10 ± 0.007 a	0.07 ± 0.015 ab	0.01 ± 0.025 b	0.02 ± 0.016 b	0.06 ± 0.020 ab	0.05 ± 0.025 ab	0.02 ± 0.018 b
C22:1	0	0	0	0.07 ± 0.115	0	0	0
C20:3 w3	0.11 ± 0.007 a	0.13 ± 0.016 a	0.18 ± 0.100 ab	0.29 ± 0.102 ab	0.19 ± 0.022 ab	0.24 ± 0.085 ab	0.59 ± 0.319 b
C20:4 w6	0.24 ± 0.056 a	0.19 ± 0.004 a	0.25 ± 0.067 a	0.20 ± 0.031 a	0.20 ± 0.008 a	0.19 ± 0.032 a	0.23 ± 0.049 a
C22-5 w3	0.08 ± 0.009 a	0.09 ± 0.019 a	0.03 ± 0.058 a	0.03 ± 0.055 a	0.09 ± 0.013 a	0.05 ± 0.046 a	0.03 ± 0.054 a
C22-6 w3	0.04 ± 0.008 ab	0.01 ± 0.018 ab	0	0.05 ± 0.038	0.02 ± 0.017	0	0
Total MUFA	44.29 ± 0.031 a	44.77 ± 0.025 b	45.77 ± 0.153 c	45.53 ± 0.132 c	45.20 ± 0.026 d	45.09 ± 0.044 d	44.40 ± 0.182 a
Omega 6 FA	16.21 ± 0.020 a	16.33 ± 0.008 ab	16.9 ± 0.141 c	16.84 ± 0.127 c	16.53 ± 0.017 b	17.1 ± 0.036 c	18.13 ± 0.171 d
Omega 3 FA	2.14 ± 0.017 a	2.12 ± 0.005 a	1.69 ± 0.138 b	2.76 ± 0.125 c	2.25 ± 0.011 a	2.29 ± 0.029 a	2.73 ± 0.162 c
Omega 6/3 FA	7.57 ± 0.011 ab	7.70 ± 0.003 b	10.00 ± 0.131 c	6.10 ± 0.120 d	7.35 ± 0.008 a	7.47 ± 0.027 ab	6.64 ± 0.161 e

a,b,c,d,e—means marked with different letters in the column differed statistically significantly (*p* < 0.05, Bonferroni criterion); SC—control sample (lean pork + back fat + ice + salt + pepper); SD10—sausages with lyophilized mealworms (lean pork + back fat + 10% mealworms + ice + salt + pepper); SD20—sausages with lyophilized mealworms (lean pork + back fat + 20% mealworms + ice + salt + pepper); SD30—sausages with lyophilized mealworms (lean pork + back fat + 30% mealworms + ice + salt + pepper); SF10—sausages with dried mealworms (lean pork + back fat + 10% mealworms + ice + salt + pepper); SF20—sausages with dried mealworms (lean pork + back fat + 20% mealworms + ice + salt + pepper); SF30—sausages with dried mealworms (lean pork + back fat + 30% mealworms + ice + salt + pepper).

**Table 6:** The amounts of biogenic amines in sausages with lyophilized larvae and dried mealworms (mg/kg of dry matter), average ± standard deviation, *n* = 3, (Average ± S.D).

	Histamine	Cadaverine	Petrescine	Tiramine	Spermine	Spermidine
SC	n.d.	n.d.	0.28 ± 0.16 a	0.25 ± 0.04 a	20.30 ± 2.66 a	2.92 ± 0.10 a
SF10	n.d.	14.46 ± 0.71 a	31.42 ± 1.38 b	1.04 ± 0.10 b	21.04 ± 1.29 a	9.97 ± 0.54 b
SF20	n.d.	cb	67.43 ± 2.87 c	2.14 ± 0.03 c	14.76 ± 0.60 b	17.09 ± 0.61 c
SF30	n.d.	14.73 ± 0.49 a	104.33 ± 2.24 d	4.48 ± 0.15 d	13.62 ± 1.74 b	26.42 ± 1.20 d
SD10	n.d.	4.55 ± 0.01 c	42.95 ± 1.03 e	1.28 ± 0.27 b	22.00 ± 0.04 a	13.28 ± 0.28 e
SD20	n.d.	4.60 ± 0.12 c	85.38 ± 3.85 f	1.66 ± 0.14 c	19.02 ± 1.03 ab	21.92 ± 1.12 f
SD30	n.d.	4.84 ± 0.10 c	121.08 ± 3.41 g	2.17 ± 0.28 c	14.98 ± 0.38 b	29.27 ± 1.12 g

a,b,c,d,e,f,g —means marked with different letters in the column differed statistically significantly (*p* < 0.05, Bonferroni criterion); SC—control sample (lean pork + back fat + ice + salt + pepper); SD10—sausages with lyophilized mealworms (lean pork + back fat + 10% mealworms + ice + salt + pepper); SD20—sausages with lyophilized mealworms (lean pork + back fat + 20% mealworms + ice + salt + pepper); SD30—sausages with lyophilized mealworms (lean pork + back fat + 30% mealworms + ice + salt + pepper); SF10—sausages with dried mealworms (lean pork + back fat + 10% mealworms + ice + salt + pepper); SF20—sausages with dried mealworms (lean pork + back fat + 20% mealworms + ice + salt + pepper); SF30—sausages with dried mealworms (lean pork + back fat + 30% mealworms + ice + salt + pepper).

## Data Availability

The original contributions presented in the study are included in the article, further inquiries can be directed to the corresponding author.

## Appendix A

**Samples/Safety Parameter**	**Nitrate, mg/kg**	**Nitrite, mg/kg**
SC	<2	<2
SF10	<2	<2
SF20	<2	<2
SF30	<2	<2
SD10	<2	<2
SD20	<2	<2
SD30	<2	<2
Mealworms	5 ± 1.00	<2
SC—control sample (lean pork + back fat + ice + salt + pepper); SD10—sausages with lyophilized mealworms (lean pork + back fat + 10% mealworms + ice + salt + pepper); SD20—sausages with lyophilized mealworms (lean pork + back fat + 20% mealworms + ice + salt + pepper); SD30—sausages with lyophilized mealworms (lean pork + back fat + 30% mealworms + ice + salt + pepper); SF10—sausages with dried mealworms (lean pork + back fat + 10% mealworms + ice + salt + pepper); SF20—sausages with dried mealworms (lean pork + back fat + 20% mealworms + ice + salt + pepper); SF30—sausages with dried mealworms (lean pork + back fat + 30% mealworms + ice + salt + pepper).		
