# Correction: Jankauskienė et al. Innovative Applications of *Tenebrio molitor* Larvae in the Production of Sustainable Meat Sausages: Quality and Safety Aspects. *Foods* 2024, *13*, 1451

**DOI:** 10.3390/foods14081376

**Published:** 2025-04-16

**Authors:** Agnė Jankauskienė, Sandra Kiseliovienė, Dominykas Aleknavičius, Ieva Miliūnaitė, Sigita Kerzienė, Žydrūnė Gaižauskaitė, Ignė Juknienė, Paulina Zaviztanavicˇiu¯ tė, Aistė Kabašinskienė

**Affiliations:** 1 Department of Food Safety and Quality, Veterinary Academy, Lithuanian University of Health Sciences, Tilzes St. 18, LT-47181 Kaunas, Lithuania; agne.jankauskiene@lsmu.lt (A.J.); ieva.miliunaite@stud.lsmu.lt (I.M.); igne.jukniene@lsmu.lt (I.J.); paulina.zavistanaviciute@lsmu.lt (P.Z.); 2 Food Institute, Kaunas University of Technology, Radvilenu pl. 19, LT-50254 Kaunas, Lithuania; sandra.kiselioviene@ktu.lt (S.K.); zydrune.gaizauskaite@ktu.lt (Ž.G.); 3 Divaks, UAB, Vinco Kudirkos g. 22-12, LT-01113 Vilnius, Lithuania; dominykas@divaks.com; 4 Department of Physics, Mathematics and Biophysics, Veterinary Academy, Lithuanian University of Health Sciences, Tilzes St. 18, LT-47181 Kaunas, Lithuania; sigita.kerziene@lsmu.lt

Due to inconsistencies identified, the authors would like to make the following corrections to this published paper [1].

The following sections originally appearing within the methods have been removed and the remaining section numbers have been modified accordingly:

- 2.3.3. A Method for Determining Color Coordinates
- 2.4.2. Method for Determination of Protein Content
- 2.4.3. Determination of Carbohydrate Content
- 2.4.4. Determination of Energy Value

Furthermore, references to the calculation formula and related computations for the above-mentioned sections have been removed throughout the publication and the related columns removed from Tables 2 and 3.

There was a typographical error in Table 2, “Physicochemical and textural properties of sausages with lyophilized and dried mealworms, average ± standard error, *n* = 3”, whereby some of the moisture content values were misaligned.

The corrected table is as follows:

**Table 2:** Physicochemical and textural properties of sausages with lyophilized and dried mealworms, average ± standard error, *n* = 3.

	pH	Cooking Loss, %	Texture Hardness, mJ	Moisture Content, %
SC	6.61 ± 0.11 a	25.94 ± 0.19 a	0.3 ± 0.020 a	42.69 ± 0.21 a
SF10	6.68 ± 0.01 a	27.37 ± 0.28 a	0.4 ± 0.050 b	36.60 ± 0.15
SF20	6.51 ± 0.02 a	21.68 ± 0.64 b	0.2 ± 0.001 c	46.42 ± 0.10 d
SF30	6.51 ± 0.08 a	16.98 ± 0.91 c	0.1 ± 0.010 d	48.07 ± 0.22 c
SD10	6.36 ± 0.09 a	21.69 ± 0.43 b	0.2 ± 0.003 c	44.59 ± 0.19 f
SD20	6.40 ± 0.12 a	19.84 ± 0.28 d	0.3 ± 0.010 a	50.00 ± 0.12 e
SD30	6.29 ± 0.82 a	15.14 ± 0.92 e	0.2 ± 0.005 c	50.22 ± 0.26 e

a,b,c,d,e,f—means marked with different letters in the column differed statistically significantly (*p* < 0.05, Bonferroni criterion); SC—control sample (lean pork + back fat + ice + salt + pepper); SD10—sausages with lyophilized mealworms (lean pork + back fat + 10% mealworms + ice + salt + pepper); SD20—sausages with lyophilized mealworms (lean pork + back fat + 20% mealworms + ice + salt + pepper); SD30—sausages with lyophilized mealworms (lean pork + back fat + 30% mealworms + ice + salt + pepper); SF10—sausages with dried mealworms (lean pork + back fat + 10% mealworms + ice + salt + pepper); SF20—sausages with dried mealworms (lean pork + back fat + 20% mealworms + ice + salt + pepper); SF30—sausages with dried mealworms (lean pork + back fat + 30% mealworms + ice + salt + pepper).

There was a typographical error in Table 3, “Chemical composition of sausages formulated with lyophilized and dried mealworm larvae, average ± standard error, *n* = 3”, whereby the salt content (%) value was incorrectly presented. The salt content value (%) has been updated to: 1.48 ± 0.01 a.

The corrected table is as follows:

**Table 3:** Chemical composition of sausages formulated with lyophilized and dried mealworm larvae, average ± standard error, *n* = 3.

	Fat, g/100 g	Ash, %	Cholesterol, mg/100 g	Hydroxyproline, g/100 g	Salt Content, %	Collagen, g/100 g
SC	23.20 ± 0.11 a	0.97 ± 0.021 a	73.86 ± 1.14 a	0.23 ± 0.010 a	1.48 ± 0.01 a	1.84 ± 0.08 a
SF10	26.55 ± 0.14 b	0.98 ± 0.002 a	64.08 ± 1.65 b	0.19 ± 0.010 bc	2.32 ± 0.10 b	1.52 ± 0.08 b
SF20	26.50 ± 0.03 b	0.98 ± 0.160 a	61.74 ± 1.25 b	0.17 ± 0.010 bd	4.06 ± 0.07 c	1.36 ± 0.08 bc
SF30	25.59 ± 0.06 c	0.98 ± 0.031 a	56.40 ± 0.98 c	0.21 ± 0.012 ac	5.8 ± 0.08 d	1.71 ± 0.09 ab
SD10	24.27 ± 0.04 d	0.98 ± 0.002 a	64.36 ± 2.11 b	0.27 ± 0.010 e	4.64 ± 0.18 e	2.16 ± 0.08 d
SD20	24.50 ± 0.04 ef	0.98 ± 0.008 a	55.96 ± 1.42 c	0.16 ± 0.006 d	5.22 ± 0.09 f	1.25 ± 0.05 c
SD30	28.65 ± 0.05	0.99 ± 0.001 a	55.65 ± 1.53 c	0.15 ± 0.010 d	6.38 ± 0.3 g	1.20 ± 0.08 c

a,b,c,d,e,f,g—means marked with different letters in the column differed statistically significantly (*p* < 0.05, Bonferroni criterion); SC—control sample (lean pork + back fat + ice + salt + pepper); SD10—sausages with lyophilized mealworms (lean pork + back fat + 10% mealworms + ice + salt + pepper); SD20—sausages with lyophilized mealworms (lean pork + back fat + 20% mealworms + ice + salt + pepper); SD30—sausages with lyophilized mealworms (lean pork + back fat + 30% mealworms + ice + salt + pepper); SF10—sausages with dried mealworms (lean pork + back fat + 10% mealworms + ice + salt + pepper); SF20—sausages with dried mealworms (lean pork + back fat + 20% meal-worms + ice + salt + pepper); SF30—sausages with dried mealworms (lean pork + back fat + 30% mealworms + ice + salt + pepper).

The wording of Section 3.2.1, the first sentence in the third paragraph, was also modified to reflect the corrections to Table 4: “The summary of the total amino acids indicates a decrease in the total amino acid content as the proportion of mealworms increases, with the lowest totals observed in the SF30 and SD30 samples, except for SF10”.

Due to a correction error the Histidine and SUM originally presented in Table 4 were incorrect. The Histidine and SUM values have been removed from Table 4 and the related values adjusted accordingly in the table and throughout the text.

The corrected table is as follows:

**Table 4:** Amino acid composition of sausages formulated with lyophilized and dried mealworm larvae, g/100 g, average ± standard error, *n* = 3.

	SC	SF10	SF20	SF30	SD10	SD20	SD30
Aspartic Acid	1.64 ± 0.029 a	1.31 ± 0.130 b	1.09 ± 0.087 bc	0.95 ± 0.032 c	2.21 ± 0.121 d	1.74 ± 0.069 a	1.00 ± 0.037 c
Glutamic acid	2.04 ± 0.039 a	1.65 ± 0.059 b	1.32 ± 0.110 c	1.12 ± 0.022 c	2.71 ± 0.133 d	2.10 ± 0.075 a	1.19 ± 0.029 c
Asparagine	n.d.	n.d.	n.d.	n.d.	n.d.	n.d.	n.d.
Serine	0.55 ± 0.021 ba	0.49 ± 0.036 b	0.41 ± 0.044 cb	0.37 ± 0.003 c	0.79 ± 0.034 d	0.63 ± 0.022 a	0.39 ± 0.008 c
Glycine	n.d.	n.d.	n.d.	n.d.	1.00 ± 0.192	0.80 ± 0.046	n.d.
Threonine	0.70 ± 0.049 ab	0.64 ± 0.036 bc	0.51 ± 0.043 cd	0.43 ± 0.010 d	1.05 ± 0.061 e	0.81 ± 0.078 a	0.46 ± 0.020 d
Arginine	0.81 ± 0.012 ac	0.65 ± 0.047 a	0.48 ± 0.014 b	0.41 ± 0.023 b	0.92 ± 0.125 c	0.80 ± 0.047 ac	0.42 ± 0.021 b
Alanine	0.72 ± 0.020 a	0.66 ± 0.031 a	0.56 ± 0.021 b	0.54 ± 0.015 b	1.07 ± 0.034 c	0.89 ± 0.040 d	0.56 ± 0.016 b
Tyrosine	0.40 ± 0.014 a	0.38 ± 0.056 a	0.31 ± 0.024 a	0.32 ± 0.021 a	0.59 ± 0.054 b	0.55 ± 0.050 b	0.37 ± 0.025 a
Cystine	1.46 ± 0.116 ab	1.07 ± 0.099 ab	0.88 ± 0.083 a	0.93 ± 0.055 a	1.16 ± 0.419 ab	1.62 ± 0.280 b	0.93 ± 0.145 a
Valine	0.40 ± 0.053 ab	0.28 ± 0.063 ab	0.19 ± 0.004 a	0.19 ± 0.043 a	0.40 ± 0.085	0.42 ± 0.124 c	0.22 ± 0.045
Methionine	n.d.	n.d.	n.d.	n.d.	n.d.	n.d.	n.d.
Tryptophan	0.16 ± 0.017 ab	0.13 ± 0.026 bc	0.08 ± 0.011 c	0.08 ± 0.017 c	0.20 ± 0.027 bc	0.16 ± 0.037 ab	0.08 ± 0.025 ab
Phenylalanine	0.59 ± 0.020 a	0.49 ± 0.028 b	0.39 ± 0.042 c	0.34 ± 0.007 c	0.76 ± 0.060 d	0.61 ± 0.015 a	0.35 ± 0.023 c
Isoleucine	0.43 ± 0.006 a	0.34 ± 0.017 b	0.27 ± 0.021 bc	0.24 ± 0.016 c	0.59 ± 0.061 d	0.47 ± 0.027 a	0.26 ± 0.018 bc
Leucine	1.02 ± 0.032 a	0.82 ± 0.046 b	0.66 ± 0.073 bc	0.58 ± 0.018 c	1.36 ± 0.117 d	1.06 ± 0.031 a	0.61 ± 0.031 c
Lysine	0.93 ± 0.014 a	0.72 ± 0.046 ab	0.44 ± 0.131 c	0.44 ± 0.016 c	1.19 ± 0.133 d	0.93 ± 0.012 a	0.48 ± 0.034 c
Proline	1.04 ± 0.107 ab	1.00 ± 0.215 ab	0.71 ± 0.152 a	1.11 ± 0.209 ab	1.95 ± 0.312 c	1.48 ± 0.078 bc	0.71 ± 0.143 a

a,b,c,d,e—means marked with different letters in the row differed statistically significantly (*p* < 0.05, Bonferroni criterion); SC—control sample (lean pork + back fat + ice + salt + pepper); SD10—sausages with lyophilized mealworms (lean pork + back fat + 10% mealworms + ice + salt + pepper); SD20—sausages with lyophilized mealworms (lean pork + back fat + 20% mealworms + ice + salt + pepper); SD30—sausages with lyophilized mealworms (lean pork + back fat + 30% mealworms + ice + salt + pepper); SF10—sausages with dried mealworms (lean pork + back fat + 10% mealworms + ice + salt + pepper); SF20—sausages with dried mealworms (lean pork + back fat + 20% mealworms + ice + salt + pepper); SF30—sausages with dried mealworms (lean pork + back fat + 30% mealworms + ice + salt + pepper).

Due to a summation error the “PUFA” originally presented in Table 5 were incorrect. Consequently, the “Total PUFA” row has been removed from Table 5 and associated references have been removed from throughout the publication. Furthermore, the “Total MUFA” values were accidently duplicated, and consequently have also been removed.

The corrected table is as follows:

**Table 5:** FA composition of sausages with lyophilized and dried mealworm larvae, average ± standard error, *n* = 3.

	SC	SF10	SF20	SF30	SD10	SD20	SD30
C10:0	0.05 ± 0.003 a	0.03 ± 0.025 a	0	0	0.02 ± 0.038 a	0.02 ± 0.019 a	0.04 ± 0.061 a
C12:0	0.06 ± 0.008 a	0.07 ± 0.026 a	0	0.12 ± 0.207 a	0.08 ± 0.036 a	0.06 ± 0.018 a	0.71 ± 1.036 a
C14:0	1.14 ± 0.014 a	1.17 ± 0.025 a	0.92 ± 0.232 a	1.42 ± 0.239 a	1.20 ± 0.048 a	1.25 ± 0.047 a	1.69 ± 0.684 a
C15:0	0.06 ± 0.006 a	0.06 ± 0.005 a	0.02 ± 0.026 a	0.03 ± 0.023 a	0.05 ± 0.010 a	0.05 ± 0.016 a	0.04 ± 0.007 a
C16:0	22.23 ± 0.281 a	22.45 ± 0.053 a	22.22 ± 0.326 a	21.98 ± 0.710 a	22.06 ± 0.030 a	22.15 ± 0.174 a	21.94 ± 0.191 a
C16:1	1.63 ± 0.009 a	1.61 ± 0.016 a	1.50 ± 0.112 a	1.52 ± 0.096 a	1.62 ± 0.031 a	1.60 ± 0.035 a	1.48 ± 0.091 a
C17:0	0.44 ± 0.013 a	0.44 ± 0.009 ab	0.32 ± 0.069 b	0.34 ± 0.043 ab	0.42 ± 0.018 ab	0.40 ± 0.031 ab	0.33 ± 0.052 ab
C17:1	0.36 ± 0.012 a	0.37 ± 0.009 a	0.26 ± 0.085 a	0.28 ± 0.041 a	0.35 ± 0.012 a	0.34 ± 0.019 a	0.28 ± 0.074 a
C18:0	13.99 ± 0.057 a	13.26 ± 0.114 d	12.69 ± 0.206 bc	12.22 ± 0.352 be	12.98 ± 0.051 cd	12.44 ± 0.030 be	12.03 ± 0.020 e
C18:1 tr.	0.18 ± 0.014 a	0.16 ± 0.016 a	0.05 ± 0.066 b	0.08 ± 0.031 ab	0.14 ± 0.022 ab	0.14 ± 0.027 ab	0.10 ± 0.031 ab
C18:1	41.56 ± 0.018 a	42.10 ± 0.101 ac	43.68 ± 0.710 b	43.02 ± 0.464 bc	42.55 ± 0.198 abc	42.52 ± 0.298 abc	42.16 ± 0.840 ac
C18:2 w6	15.22 ± 0.239 a	15.48 ± 0.028 ab	16.26 ± 0.606 ab	16.19 ± 0.574 ab	15.69 ± 0.068 ab	16.33 ± 0.249 ab	16.70 ± 0.697 b
C20:0	0.18 ± 0.014 a	0.15 ± 0.026 ab	0.03 ± 0.044 a	0.03 ± 0.043 a	0.13 ± 0.036 ab	0.12 ± 0.058 ab	0.08 ± 0.079 ab
C18:3 α w3	0.90 ± 0.004 a	0.88 ± 0.010 a	0.65 ± 0.196 a	1.05 ± 0.012 a	0.88 ± 0.016 a	0.88 ± 0.010 a	0.76 ± 0.129 a
C20:1	0.74 ± 0.030 a	0.69 ± 0.039 a	0.33 ± 0.305 a	0.64 ± 0.120 a	0.68 ± 0.015 a	0.63 ± 0.034 a	0.48 ± 0.129 a
C18:3 ґ w6	0.03 ± 0.010 a	0.01 ± 0.020 a	0	0.01 ± 0.415 a	0.01 ± 0.017 a	0.01 ± 0.034 a	0.76 ± 0.134 a
C21:0	0.04 ± 0.004 a	0.02 ± 0.028 a	0.21 ± 0.367 a	0	0	0.01 ± 0.021 a	0
C20:2 w6	0.62 ± 0.005 a	0.58 ± 0.016 ac	0.38 ± 0.119	0.42 ± 0.057 bc	0.57 ± 0.028 abc	0.52 ± 0.039 abc	0.42 ± 0.092 b
C22:0	0.01 ± 0.012	0	0	0	0	0	0
C20:3 w6	0.10 ± 0.007 a	0.07 ± 0.015 ab	0.01 ± 0.025 b	0.02 ± 0.016 b	0.06 ± 0.020 ab	0.05 ± 0.025 ab	0.02 ± 0.018 b
C22:1	0	0	0	0.07 ± 0.115	0	0	0
C20:3 w3	0.11 ± 0.007 a	0.13 ± 0.016 a	0.18 ± 0.100 ab	0.29 ± 0.102 ab	0.19 ± 0.022 ab	0.24 ± 0.085 ab	0.59 ± 0.319 b
C20:4 w6	0.24 ± 0.056 a	0.19 ± 0.004 a	0.25 ± 0.067 a	0.20 ± 0.031 a	0.20 ± 0.008 a	0.19 ± 0.032 a	0.23 ± 0.049 a
C22-5 w3	0.08 ± 0.009 a	0.09 ± 0.019 a	0.03 ± 0.058 a	0.03 ± 0.055 a	0.09 ± 0.013 a	0.05 ± 0.046 a	0.03 ± 0.054 a
C22-6 w3	0.04 ± 0.008 ab	0.01 ± 0.018 ab	0	0.05 ± 0.038	0.02 ± 0.017	0	0
Total MUFA	44.29 ± 0.031 a	44.77 ± 0.025 b	45.77 ± 0.153 c	45.53 ± 0.132 c	45.20 ± 0.026 d	45.09 ± 0.044 d	44.40 ± 0.182 a
Omega 6 FA	16.21 ± 0.020 a	16.33 ± 0.008 ab	16.9 ± 0.141 c	16.84 ± 0.127 c	16.53 ± 0.017 b	17.1 ± 0.036 c	18.13 ± 0.171 d
Omega 3 FA	2.14 ± 0.017 a	2.12 ± 0.005 a	1.69 ± 0.138 b	2.76 ± 0.125 c	2.25 ± 0.011 a	2.29 ± 0.029 a	2.73 ± 0.162 c
Omega 6/3 FA	7.57 ± 0.011 ab	7.70 ± 0.003 b	10.00 ± 0.131 c	6.10 ± 0.120 d	7.35 ± 0.008 a	7.47 ± 0.027 ab	6.64 ± 0.161 e

a,b,c,d,e—means marked with different letters in the column differed statistically significantly (*p* < 0.05, Bonferroni criterion); SC—control sample (lean pork + back fat + ice + salt + pepper); SD10—sausages with lyophilized mealworms (lean pork + back fat + 10% mealworms + ice + salt + pepper); SD20—sausages with lyophilized mealworms (lean pork + back fat + 20% mealworms + ice + salt + pepper); SD30—sausages with lyophilized mealworms (lean pork + back fat + 30% mealworms + ice + salt + pepper); SF10—sausages with dried mealworms (lean pork + back fat + 10% mealworms + ice + salt + pepper); SF20—sausages with dried mealworms (lean pork + back fat + 20% mealworms + ice + salt + pepper); SF30—sausages with dried mealworms (lean pork + back fat + 30% mealworms + ice + salt + pepper).

The wording of Section 3.3.2, the 6th to 7th sentences, was also modified to reflect the corrections to Table 5: “However, none of the tested samples containing mealworms exceeded the recommended limit. This limitation may be attributed to the distinct chemical compositions observed between brewers’ spent grain and alternative growth mediums, potentially affecting the chemical reactions and metabolic processes within the larvae [14]”.

The authors state that the scientific conclusions are unaffected. This correction was approved by the Academic Editor. The original publication has also been updated.
